# “The pivotal role of proper nasolabial angle management in the functional treatment of skeletal Class II malocclusion: an opinion article”

**DOI:** 10.3389/froh.2026.1787760

**Published:** 2026-03-02

**Authors:** Emma Gotti, Antonio Manni, Giorgio Gastaldi, Mauro Cozzani, Andrea Boggio

**Affiliations:** 1 Department of Dentistry, Vita-Salute San Raffaele University, Milan, Italy; 2 Istituto Giuseppe Cozzani, La Spezia, Italy

**Keywords:** Class II malocclusion, functional appliances, Herbst, nasolabial angle, skeletal anchorage

## 1 Introduction

Class II malocclusion is most commonly characterized by mandibular retrusion (1). To address this condition, orthodontic treatment has traditionally relied on functional appliances, which aim to advance the mandible, improve occlusal relationships, and straighten the facial profile. However, it is well documented that functional therapy is associated with intrinsic side effects related to anchorage loss, including proclination of the lower incisors and retroclination of the upper incisors (2, 3). While these effects are often considered secondary to skeletal correction, their impact on soft tissue profile may be clinically significant.

The retroclination of the maxillary incisors is particularly critical, as it not only limits effective mandibular advancement but also increases the nasolabial angle (NLA), potentially compromising facial aesthetics. While extensive attention has been devoted to skeletal effects—especially pogonion advancement—changes in the nasolabial angle following functional treatment of Class II malocclusion remain relatively underexplored.

In addition to orthodontic mechanics, physiological nasal growth plays a relevant role in determining changes in the nasolabial angle. During adolescence and early adulthood, the nose undergoes continuous growth characterized by anterior and inferior projection of the nasal tip, as well as changes in columellar inclination. These modifications may alter the nasolabial angle independently of orthodontic treatment. Longitudinal investigations have demonstrated that nasal growth continues beyond the pubertal growth spurt and may contribute to progressive variations in soft tissue profile (4, 5).

Therefore, when evaluating changes in the nasolabial angle during Class II treatment—particularly in growing patients—it is essential to distinguish between treatment-induced dentoalveolar effects and physiological soft tissue maturation.

This opinion article argues that the nasolabial angle deserves greater consideration in the orthodontic management of skeletal Class II malocclusion, particularly when functional appliances are used.

## 2 Subsections relevant to the subject

### 2.1 The nasolabial angle and orthodontic treatment

**Figure 1:**
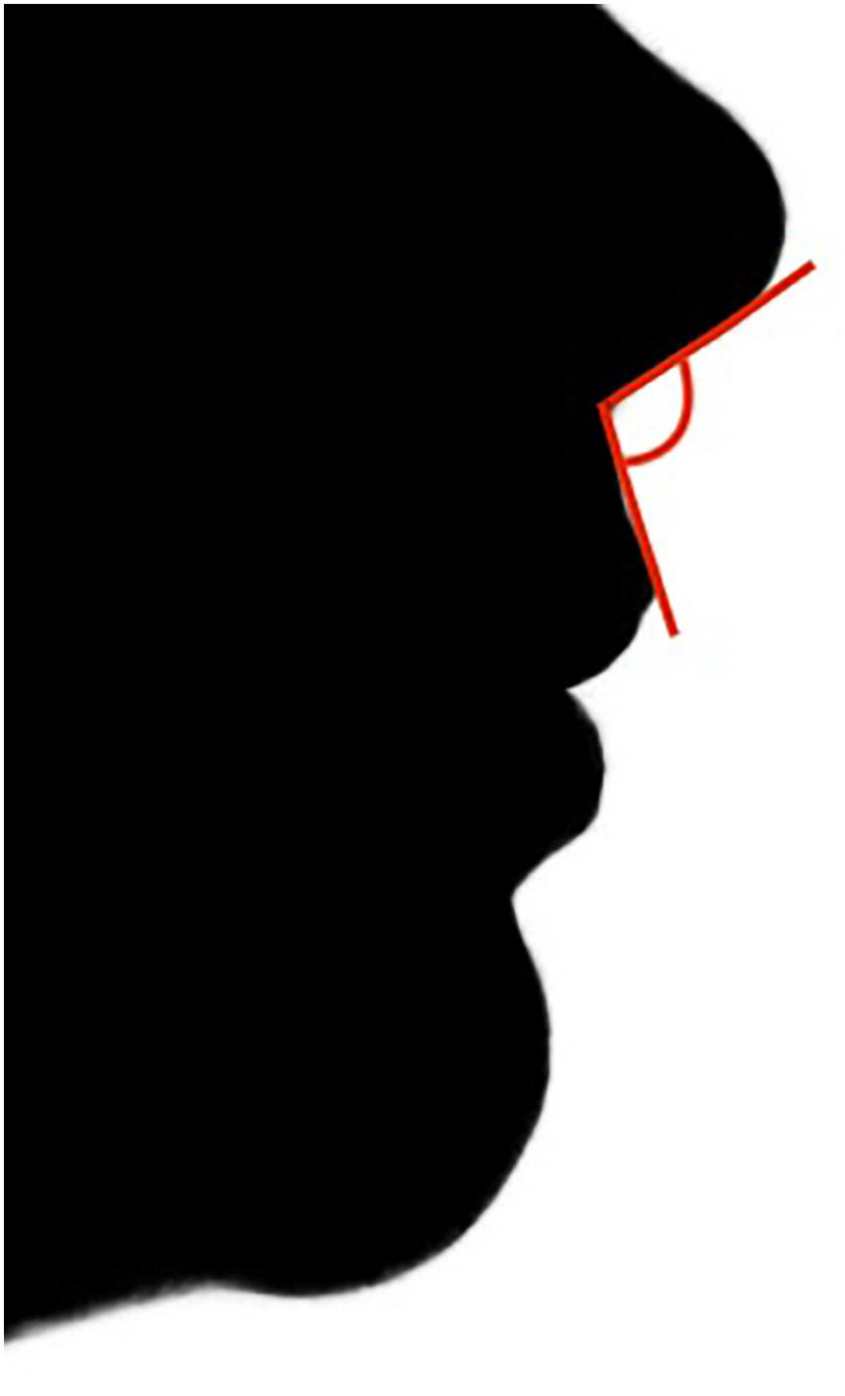
Schematic representation of the nasolabial angle (NLA). The nasolabial angle is formed by the intersection of a line tangent to the columella and a line along the upper lip. Variations in nasal growth and maxillary incisor position may influence its magnitude.

The nasolabial angle is formed by the intersection of a line tangent to the columella and a line along the upper lip. Normative values vary by sex, with women typically presenting more obtuse angles (100–105°) and men more acute angles (90–95°) (6). The nasolabial angle (NLA) is schematically illustrated in Figure 1.

Beyond nasal base inclination, the NLA is strongly influenced by upper lip prominence and maxillary incisor position, making it highly sensitive to orthodontic tooth movement.

Several studies have investigated the effects of orthodontic camouflage treatments—such as premolar extractions and maxillary distalization—on facial profile and the nasolabial angle. As early as the 1980s, Lo and colleagues demonstrated that maxillary incisor retroclination was associated with a significant increase in the NLA when comparing treated and untreated individuals.

While no relevant changes were observed in untreated subjects during growth, the treated group exhibited a clear correlation between incisor retraction and angle opening: on average, each millimeter of maxillary incisor retraction resulted in an increase of approximately 1.63° in the nasolabial angle (7). Similar findings have been reported in extraction-based treatment protocols (8).

These data suggest that dentoalveolar mechanics may significantly influence facial aesthetics, even in the absence of skeletal changes.

Moreover, it should be emphasized that Class II malocclusion is a heterogeneous condition that may result from different skeletal and dentoalveolar patterns, including maxillary protrusion, mandibular retrusion, dental compensations, vertical discrepancies, and, in some cases, altered cranial base morphology. In addition, variations in nasal growth and facial development may further influence the soft tissue profile. Diagnosis and evaluation of treatment effectiveness are commonly based on cephalometric parameters such as the ANB angle, overjet (OJ), and assessment of the spatial relationship between the maxilla and mandible (9). While these measurements are essential for evaluating sagittal correction, they do not fully capture changes occurring in the nasolabial region.

### 2.2 Functional appliances and dentoalveolar compensation

Currently, functional appliances represent the treatment of choice for skeletal Class II malocclusions due to their documented ability to promote mandibular advancement. This forward repositioning improves occlusal relationships—often achieving a Class I molar relationship—and reduces excessive overjet (10).

Nevertheless, their effects are not purely skeletal. Anchorage loss frequently results in retroclination of maxillary incisors and proclination of mandibular incisors (2). While mandibular incisor proclination may reduce the available sagittal advancement, maxillary incisor retroclination may lead to increased nasolabial angle opening.

Despite these clinically relevant soft tissue implications, most studies on functional appliances have focused primarily on skeletal landmarks, particularly changes in pogonion position, with limited and sometimes conflicting evidence regarding modifications of the nasolabial angle (11–13).

Booij et al. compared Herbst therapy with premolar extraction treatment and observed an increase in the NLA in both groups, although the increase was greater in the extraction group (mean 2.3°) compared with the Herbst group (mean 0.7°) (14). Some years later, analogous results werealso reported by Janson et al., who likewise compared Herbst therapy with extraction treatment (15). Although less pronounced, the increase observed with the Herbst appliance remains clinically relevant, particularly in patients with severe overjet, in whom dentoalveolar compensations tend to be more marked.

### 2.3 Treatment strategies to control dentoalveolar compensation and preserve the nasolabial angle

Different treatment strategies may be adopted to minimize dentoalveolar side effects during functional correction of Class II malocclusion and thereby preserve the nasolabial angle. From a biomechanical perspective, the key objective is to control maxillary incisor retroclination and mandibular incisor proclination, independently of the specific appliance used.

To reduce dentoalveolar side effects, functional appliances are increasingly combined with skeletal anchorage. However, when temporary anchorage devices (TADs) are placed exclusively in the mandibular arch to control lower incisor proclination, reactive forces may be redirected to the maxillary dentition, potentially exacerbating maxillary incisor retroclination.

For this reason, simultaneous anchorage control in both arches appears biomechanically more consistent (16). Miniplate-supported protocols have been proposed, either by anchoring functional appliances directly to mandibular miniplates (17), or by supporting Class II elastics with miniplates in both arches (18).

Although effective, these approaches are relatively invasive and may not be routinely applicable.

A more conservative alternative involves the use of miniscrews as indirect skeletal anchorage combined with functional appliances (19–23).

One possible clinical application of this strategy involves the use of a functional appliance reinforced with miniscrews in both arches. For example, a Herbst appliance incorporating a fixed transpalatal bar and a lower acrylic splint can be reinforced with four orthodontic miniscrews—two in the mandible and two in the maxilla—connected via elastic ligatures delivering approximately 150–200 g of force.

Mandibular miniscrews, placed in interradicular vestibular areas, limit lower incisor proclination in synergy with the acrylic splint. Maxillary miniscrews, whether placed palatally or vestibularly depending on treatment objectives, help prevent upper incisor retroclination and thus contribute to preservation of the nasolabial angle (22–25).

By improving anchorage control, this strategy may allow sagittal correction while minimizing unfavorable soft tissue changes.

## 3 Discussion

Orthodontic treatment of skeletal Class II malocclusion has traditionally prioritized skeletal correction and occlusal relationships. However, facial aesthetics cannot be reduced to sagittal mandibular advancement alone.

The nasolabial angle represents a key parameter in the lower third of the face and is particularly sensitive to maxillary incisor positioning. Excessive retroclination, even when secondary to functional therapy, may result in an undesirable flattening of the upper lip profile.

This article does not advocate for a specific appliance or protocol. Rather, it proposes that greater attention should be devoted to the nasolabial region when planning and monitoring functional treatment. Skeletal anchorage—especially when applied in both arches—may represent a biomechanically sound approach to limit dentoalveolar compensation and preserve facial harmony.

Future research should further clarify the magnitude and clinical perception of nasolabial angle changes associated with different treatment modalities. Until then, clinicians should integrate soft tissue considerations into their decision-making process, ensuring that sagittal correction does not come at the expense of facial aesthetics.

From a treatment-planning perspective, strategies that prioritize sagittal correction while simultaneously controlling maxillary incisor position may be more favorable in preserving the nasolabial angle. Approaches that reduce anchorage loss—particularly through bilateral skeletal anchorage control—may limit undesirable dentoalveolar compensation and soft tissue flattening. Conversely, strategies primarily based on dental camouflage or uncontrolled incisor retraction may carry a greater risk of excessive nasolabial angle opening.

Therefore, optimal management of skeletal Class II malocclusion should integrate sagittal skeletal objectives with careful soft tissue monitoring, ensuring that functional correction does not compromise facial harmony.

## References

[1] McNamara JA. Components of class II malocclusion in children 8–10 years of age. Angle Orthodontist. (1981) 51(3):177–202. 10.1043/0003-3219(1981)051<0177:COCIMI>2.0.CO;2 7023290

[2] Perinetti G Primožič J Franchi L Contardo L. Treatment effects of removable functional appliances in Pre-pubertal and pubertal class II patients: a systematic review and meta-analysis of controlled studies. PLoS One. (2015) 10(10). 10.1371/journal.pone.0141198 26510187 PMC4624952

[3] Cozza P Baccetti T Franchi L Toffol LD McNamara JA. Mandibular changes produced by functional appliances in class II malocclusion: a systematic review. Am J Orthod Dentofacial Orthop. (2006) 129(5). Preprint. 10.1016/j.ajodo.2005.11.010 16679196

[4] Baddam P Bayona-Rodriguez F Campbell SM El-Hakim H Graf D. Properties of the nasal cartilage, from development to adulthood: a scoping review. Cartilage. (2022) 13(1). 10.1177/19476035221087696 35345900 PMC9137313

[5] Saniasiaya J Abdullah B. Critical review of the literature on conventional septoplasty in children. B-ENT. (2021) 17(3). 10.5152/B-ENT.2021.21533

[6] Sinno HH Markarian MK Ibrahim AMS Lin SJ. The ideal nasolabial angle in rhinoplasty: a preference analysis of the general population. Plast Reconstr Surg. (2014) 134(2). 10.1097/PRS.0000000000000385 25068320

[7] Lo FD Hunter WS. Changes in nasolabial angle related to maxillary incisor retraction. Am J Orthod. (1982) 82(5). 10.1016/0002-9416(82)90187-7 6961809

[8] Freitas DB Lotif MAL FO C. Changes in facial aesthetics arising from dental retraction in class II maloclusions division 1. Oral Health Dent Manag. (2020) 19(3):1–7.

[9] Danz JC Stöckli S Rank CP. Precision and accuracy of craniofacial growth and orthodontic treatment evaluation by digital image correlation: a prospective cohort study. Front Oral Health. (2024) 5. 10.3389/froh.2024.1419481 39130491 PMC11310159

[10] Pancherz H. The herbst appliance-its biologic effects and clinical use. Am J Orthod. (1985) 87(1). 10.1016/0002-9416(85)90169-1 3855346

[11] D’Antò V Bucci R Franchi L Rongo R Michelotti A Martina R. Class II functional orthopaedic treatment: a systematic review of systematic reviews. J Oral Rehabil. (2015) 42(8). Preprint. 10.1111/joor.12295 25824331

[12] Flores-Mir C Major PW. Cephalometric facial soft tissue changes with the twin block appliance in class II division 1 malocclusion patients: a systematic review. Angle Orthodontist. (2006) 76(5). Preprint. 10.1043/0003-3219(2006)076[0876:CFSTCW]2.0.CO;2 17029526

[13] Flores-Mir C Major MP Major PW. Soft tissue changes with fixed functional appliances in class II division 1: a systematic review. Angle Orthodontist. (2006) 76(4):712–9. 10.1043/0003-3219(2006)076[0712:STCWFF]2.0.CO;2 16808582

[14] Booij JW Goeke J Bronkhorst EM Katsaros C Ruf S. Class II treatment by extraction of maxillary first molars or herbst appliance: dentoskeletal and soft tissue effects in comparison. J Orofacial Orthop. (2013) 74(1). 10.1007/s00056-012-0112-1 23299649

[15] Janson G Castello Branco N Del Castillo AA Henriques JFC Fernandes De Morais J. Soft tissue treatment changes with fixed functional appliances and with maxillary premolar extraction in class II division 1 malocclusion patients. Eur J Orthod. (2018) 40(2). Preprint. 10.1093/ejo/cjx053 29016727

[16] Gotti E Doldo T Castellana F Manni A Gastaldi G Cozzani M Mandibular advancement and skeletal anchorage in class II malocclusion patients: a systematic review with meta-analysis. Oral. (2024) 4(3). Preprint. 10.3390/oral403003440330111

[17] Manzo P Martina S Leone P D’antò V. Functional class II treatment with a miniplate-anchored herbst appliance. J Clin Orthod. (2021) 55(4). 34133328

[18] Al-Dumaini AA Halboub E Alhammadi MS Ishaq RAR Youssef M. A novel approach for treatment of skeletal class II malocclusion: miniplates-based skeletal anchorage. Am J Orthod Dentofacial Orthop. (2018) 153(2). 10.1016/j.ajodo.2017.06.020 29407501

[19] Al-Dboush R Soltan R Rao J El-Bialy T. Skeletal and dental effects of herbst appliance anchored with temporary anchorage devices: a systematic review with meta-analysis. Orthodontics Craniofacial Res. (2022) 25(1). 10.1111/ocr.12510 34145968

[20] Huang Y Sun W Xiong X Zhang Z Liu J Wang J. Effects of fixed functional appliances with temporary anchorage devices on class II malocclusion: a systematic review and meta-analysis. J World Fed Orthod. (2021) 10(2). Preprint. 10.1016/j.ejwf.2021.02.001 33785320

[21] Elkordy SA Aboelnaga AA Salah Fayed MM Aboulfotouh MH Abouelezz AM. Can the use of skeletal anchors in conjunction with fixed functional appliances promote skeletal changes? A systematic review and meta-analysis. Eur J Orthod. (2016) 38(5). Preprint. 10.1093/ejo/cjv081 26715339

[22] Manni A Boggio A Castellana F Gastaldi G Cozzani M. Mandibular advancement after pubertal peak with acrylic splint herbst appliance anchored to four miniscrews: a retrospective controlled study. Oral. (2024) 4(4). 10.3390/oral404003640330111

[23] Manni A Pera S Gastaldi G Boggio A Cozzani M. Skeletal anchorage in treating skeletal class II malocclusion in growing patients using the herbst appliance. Oral. (2023) 3(4). 10.3390/oral304004438162993

[24] Manni A Migliorati M Calzolari C Silvestrini-Biavati A. Herbst appliance anchored to miniscrews in the upper and lower arches vs standard herbst: a pilot study. Am J Orthod Dentofacial Orthop. (2019) 156(5). 10.1016/j.ajodo.2018.11.015 31677670

[25] Manni A Lupini D Cozzani M. Four TADs supported herbst mechanics: a case report. Int Orthodontics. (2019) 17(2). 10.1016/j.ortho.2019.03.018 31036464

